# Pathogenesis and Current Treatment of Osteosarcoma: Perspectives for Future Therapies

**DOI:** 10.3390/jcm10061182

**Published:** 2021-03-12

**Authors:** Richa Rathore, Brian A. Van Tine

**Affiliations:** 1 Division of Medical Oncology, Washington University in St. Louis, St. Louis, MO 63110, USA; richarathore@wustl.edu; 2 Division of Pediatric Hematology and Oncology, St. Louis Children’s Hospital, St. Louis, MO 63110, USA; 3 Siteman Cancer Center, St. Louis, MO 63110, USA

**Keywords:** osteosarcoma, mesenchymal stem cell, osteoblast, sarcoma, methotrexate

## Abstract

Osteosarcoma is the most common primary malignant bone tumor in children and young adults. The standard-of-care curative treatment for osteosarcoma utilizes doxorubicin, cisplatin, and high-dose methotrexate, a standard that has not changed in more than 40 years. The development of patient-specific therapies requires an in-depth understanding of the unique genetics and biology of the tumor. Here, we discuss the role of normal bone biology in osteosarcomagenesis, highlighting the factors that drive normal osteoblast production, as well as abnormal osteosarcoma development. We then describe the pathology and current standard of care of osteosarcoma. Given the complex heterogeneity of osteosarcoma tumors, we explore the development of novel therapeutics for osteosarcoma that encompass a series of molecular targets. This analysis of pathogenic mechanisms will shed light on promising avenues for future therapeutic research in osteosarcoma.

## 1. Introduction

Osteosarcomas are the most common pediatric and adult bone tumor, with more than 1000 new cases every year in the United States alone. Osteosarcomas arise from mesenchymal cells and are characterized by areas of abnormal bone growth [1]. The various genetic, epigenetic, and environmental factors that drive mesenchymal stem cells to differentiate into bone precursor cells also play a role in the development of osteosarcoma. These molecular pathways can serve as the foundation for the development of new therapies for this tumor [2]. This review describes the basic biology of bone, and how the systems that drive bone development lead to osteosarcomagenesis. Furthermore, the cellular pathways that contribute to the pathogenesis of the tumor are explored, and this information is used to describe the avenues for novel treatment development for osteosarcoma.

## 2. Bone Biology

Bone consists of four major cell types: osteoblasts, osteoclasts, osteocytes, and bone-lining cells. The bone microenvironment also includes the cartilage surrounding the bone, which consists of chondrocytes, the endothelial cells and fibroblasts that make up the bone stroma, as well as bone marrow-derived hematopoietic and mesenchymal stem cells [3,4,5]. Mesenchymal stem cells are the precursor to osteoblasts and osteocytes, as well as fibroblasts and chondrocytes.

**Figure 1:**
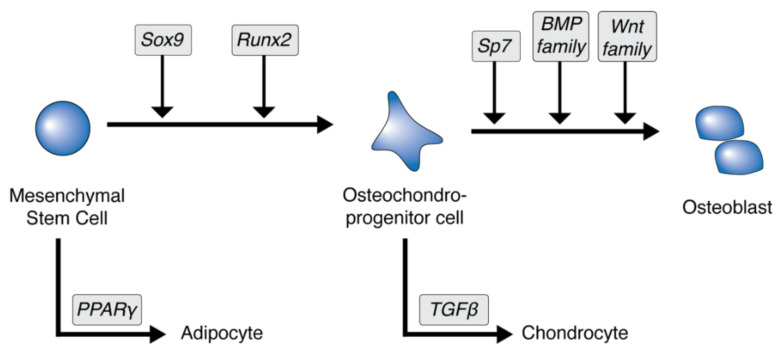
Mesenchymal stem cells differentiate into various cell types based on the expression of different transcription factors and protein families, highlighted in gray. Sox9: SRY-box transcription factor 9; Runx2: Runt-related transcription factor 2; Sp7: osterix; BMP: bone morphogenic protein; Wnt: wingless and int-1; PPARγ: peroxisome proliferator-activated receptor gamma; TGFβ: transforming growth factor-beta.

Stem cells differentiate in response to the expression and absence of various transcription factors [6]. For example, peroxisome proliferator-activated receptor gamma (PPARγ) drives the differentiation of mesenchymal stem cells into adipocytes, while Runt-related transcription factor 2 (Runx2) and sex determining region Y (SRY)-box transcription factor 9 (SOX9) drive differentiation into osteochondroprogenitor cells (Figure 1) [7]. These cells control the formation of osteoblasts and bone matrix and recruit hematopoietic cells to incorporate blood vessels. Transforming growth factor-beta (TGFβ) expression can further drive differentiation into chondrocytes, but also stimulates alkaline phosphatase (ALP) activity and calcium deposition [1,7].

Runx2 is a transcription factor that drives the expression of a series of genes related to osteogenesis [8]. Runx2 increases the expression of osterix (Sp7), which is required to commit osteochondroprogenitor cells to osteoblast differentiation, as well as osteocalcin, Type I collagen, and ALP to stimulate osteoblast formation [9,10,11]. Finally, Runx2 induces the expression of the CDK2 inhibitor p27KIP1, which coordinates G1 cell-cycle arrest in osteoblasts, a process necessary for normal development of bone. Importantly, the expression of Runx2 and ALP decreases as cells differentiate into osteoblasts, and low Runx2 expression is required for normal osteoblast function [8]. 

Bone morphogenic proteins (BMPs) comprise a family of over 30 different proteins, including TGFβ family members, that also regulate mesenchymal stem cell differentiation into osteoblasts by activating and inhibiting several genes that affect the expression of Runx2 and Sp7 [9]. Wingless and int-1 (Wnt) signaling proteins and fibroblast growth factors (FGF) further contribute to this regulation [7,9]. 

Once osteoblasts have been derived, they coordinate with osteoclasts to model and remodel bone, thereby maintaining bone homeostasis. Osteoblasts are the bone-forming cells, which create cartilage using calcium which is then hardened into bone. Osteoclasts, which are derived from hematopoietic stem cells, are the bone-resorbing cells, which break down bone using electrolytes and bone-degrading enzymes. During development, the body models bone by removing bone from some areas and synthesizing bone in others. Once development is completed, this process is termed remodeling, as the bone physically maintains its location but is constantly regenerated. 

Receptor activator of nuclear-factor kappa B (RANK) ligand (RANKL) dictates when deposited bone must be resorbed. RANKL presents on the surfaces of osteoblasts and stromal cells and binds the RANK protein on the surface of osteoclasts. Through the varied expression of RANKL, osteoblasts can control osteoclast differentiation and bone resorption [4]. Increased calcium levels can also stimulate osteoclast activity and contribute to bone resorption [3]. Osteoclasts couple with osteoblasts in a negative feedback loop in order to regulate bone homeostasis, secreting factors that both inhibit osteoclast activity and provide substrates for osteoblast activity [12].

## 3. Bone Transformation to Osteosarcoma

### 3.1. Bone Cancer and Sarcoma

Cancer is defined as a disease of abnormal cells that acquire certain capabilities that drive unchecked, uncontrolled, and invasive growth and division [13,14]. Cancers are grouped into several categories, including carcinomas, sarcomas, myelomas, leukemias, and lymphomas. Carcinomas, which arise from epithelial cells, comprise approximately 90% of human cancers. Sarcomas, which have a mesenchymal cell of origin, consist of only 1% of adult cancers. Given that the bone consists of a series of cell types that originate from mesenchymal stem cells, the tumors that arise in bone all fall into the sarcoma category [15].

There are a series of other bone sarcomas, including Ewing’s sarcoma, chondrosarcoma, hemangiosarcoma, giant cell tumor, chordoma, and the soft tissue sarcomas of bone [15,16]. There are approximately 3600 new bone cancer cases every year [17]. Osteosarcomas are the most common bone tumor, consisting of 40–50% of bone sarcomas.

### 3.2. Osteosarcoma Cell of Origin

**Figure 2:**
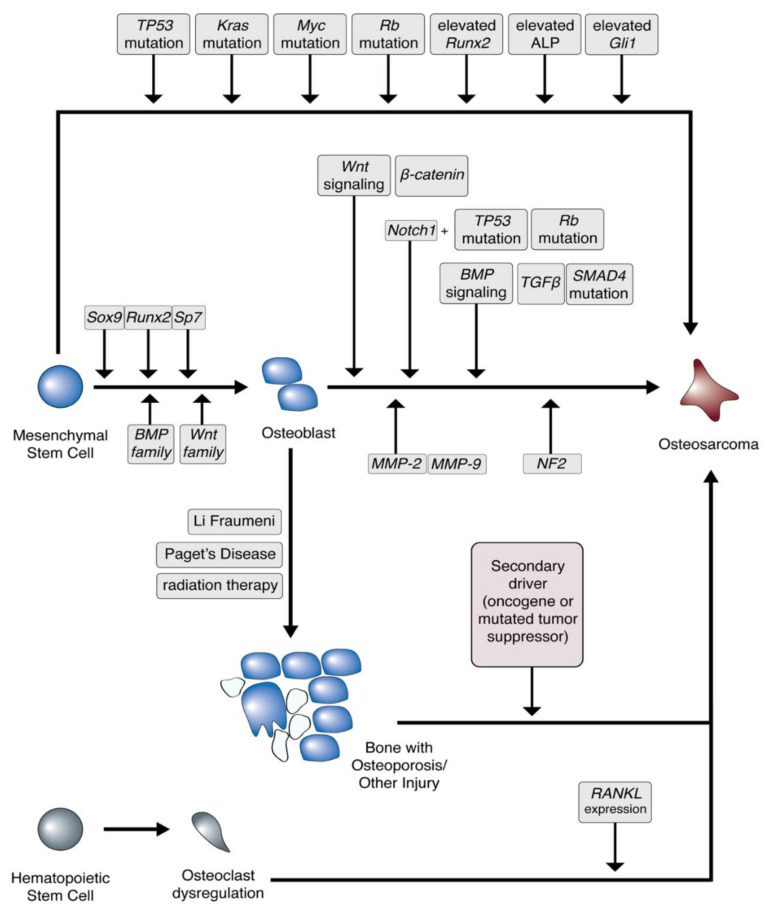
Overexpression of certain transcription factors and oncogenes and dysregulation of tumor suppressor genes can drive osteosarcoma development. TP53: tumor protein p53; Rb: retinoblastoma; Runx2: Runt-related transcription factor 2; ALP: alkaline phosphatase; Gli1: glioma-associated oncogene homolog 1; Sox9: SRY-box transcription factor 9; Sp7: osterix; BMP: bone morphogenic protein; Wnt: wingless and int-1; TGFβ: transforming growth factor-beta; SMAD4: Mothers against decapentaplegic homolog 4; MMP: matrix metalloproteinase; NF2: neurofibromatosis-2; RANKL: receptor activator of nuclear-factor kappa B ligand.

Osteosarcomagenesis was originally classified as occurring only from mesenchymal stem cells, though more recent data suggest that osteosarcomas can form at multiple points in bone development, from both mesenchymal stem cells and osteoblasts, as well as dysregulated osteoclasts (Figure 2) [18,19,20]. Unlike many other sarcomas which are driven by genetic translocations, such as synovial sarcoma or Ewing’s sarcoma, osteosarcomas have complex karyotypes [6,21]. Even so, it is widely understood that alterations to TP53 and RB1 tumor suppressor genes play a role in osteosarcoma, as in the development of several other cancers [3,22]. It has also been demonstrated that, once committed to the osteogenic lineage, MSCs with p53 and Rb excised develop into osteosarcoma-like tumors, further demonstrating the oncogenic potential of mutations to these genes [23]. 

Genes that relate to osteoblast development have also been associated with osteosarcomagenesis (Figure 2). Wnt protein family members have been identified as playing a significant role in the development of osteoblasts from mesenchymal stem cells [9,24]. Aberrant activation of Wnt family members can drive the further progression of osteoblasts into osteosarcoma. In fact, β-catenin, a mediator of Wnt family signaling, has been demonstrated to be expressed in a large percentage of osteosarcoma tumors [25].

BMP/TGFβ family members that drive osteoblast development can also drive osteosarcoma development. Interestingly, osteosarcoma tumors tend to express higher amounts of TGFβ1 and TGFβ3, which have been associated with disease progression [6]. TGFβ also activates SMAD proteins, which can inhibit osteoblast differentiation by decreasing the expression of osteocalcin [26,27]. Smad4 gene mutations have been identified in several cancers, including pancreatic and ovarian cancer, and SMAD proteins have also been identified as being dysregulated in osteosarcoma [28].

The elevated expression of Runx2, one of the main drivers of osteoblast formation from osteochondroprogenitor cells through the coordinated activation of osteocalcin, Type I collagen, and ALP, has been shown to drive osteosarcomagenesis [10,29]. Runx2 has been shown to physically interact with and be regulated by Rb and Myc, further demonstrating the complicated interactions that drive dysregulation of normal development into osteosarcoma [10]. Importantly, p27KIP1 is lost in differentiated osteosarcoma, driving cell cycle exit and normal bone development upon Runx2-mediated activation [6]. ALP, another factor elevated by Runx2, is required for the differentiation of mesenchymal stem cells to osteoblasts, but decreases as normal differentiation continues [1]. Enhanced serum ALP levels have been identified in osteosarcoma patients, indicating a role in osteosarcomagenesis [30,31]. Given the contribution of Runx2 and downstream factors to osteosarcomagenesis and bone development, it is therefore consistent that elevated Runx2 expression has been correlated with significantly poorer outcomes in osteosarcoma [32].

Gli1, an oncogene that drives the sonic hedgehog (Shh) signaling pathway, has been shown to enhance osteoblast differentiation from mesenchymal stem cells. In addition, Gli1-expressing embryonic cells have been identified as precursors to osteoblasts in mice [33]. Gli1 expression has also been shown to drive osteosarcoma development and has been associated with enhanced tumorigenesis, along with other members of the Shh signaling pathway [10,34].

Several additional pathways may also contribute to the enhanced capacity for migration and invasion and the common incidence of pulmonary metastases in osteosarcoma. The ERK1/2 pathway has been demonstrated to be crucial for migration and invasion in osteosarcoma [35]. Matrix metalloproteinases (MMPs) are a protein family that are required for the degradation of extracellular matrix proteins, which is a process that is critical for the migration and invasion of cancers. MMP-2 and MMP-9 have been demonstrated to be overexpressed in osteosarcoma and promote lung metastasis, and regulation of these enzymes is associated with other metabolic pathways that are overexpressed in osteosarcoma, including de novo serine biosynthesis [6,10,36,37]. Finally, alterations to neurofibromatosis-2 (NF2) have been correlated with increased incidence of several highly metastatic tumors, including osteosarcoma [6]. The protein encoded for by NF2, Merlin, has been demonstrated to stabilize p53; therefore, in patients with NF2 alterations, p53 is also affected, and can thereby drive incidence and malignancy of osteosarcoma [6,10].

### 3.3. Bone Microenvironment

The signaling components of the bone microenvironment play a critical role in osteosarcoma development. BMP2 and TGFβ circulate throughout the bone microenvironment, contributing to osteoblast formation but also osteosarcoma differentiation and malignancy [38,39]. Growth-related factors can also contribute to sarcomagenesis as these factors are necessary for osteoblast-driven bone formation [40]. Chondrocytes secrete high-mobility group box 1 protein (HMGB1) that stimulates osteoblast proliferation and can induce osteosarcoma proliferation [3,41]. 

Factors secreted throughout the bone microenvironment also contribute to abnormal osteoclast activity, which can result in osteosarcoma. As previously noted, osteoclasts are regulated by RANK signaling, which is mediated by RANKL expression on osteoblasts. Dysregulation of RANKL expression and ligand binding by osteoblasts and other cells in the bone microenvironment can limit bone resorption by osteoclasts and allows bone to form unchecked. Factors released by cancer cells including interleukins (IL) such as IL-6 and IL-11, as well as TGFβ, can also modulate RANK expression on the osteoclast surface that can further decrease bone resorption and contribute to tumor progression [3].

In addition to being the precursor for osteoblasts, chondrocytes, and osteosarcoma, mesenchymal stem cells themselves also play a role in tumor progression. The cytokines secreted by mesenchymal stem cells in the bone microenvironment, including TGFβ and tumor necrosis factor α (TNFα), can inhibit lymphocyte proliferation and block the response of the immune system, allowing the tumor to escape the inflammatory response [42]. Mesenchymal stem cells can also promote angiogenesis through differentiation into fibroblasts and producing growth factors, thereby improving blood supply to the tumor [43]. Finally, various factors released from mesenchymal stem cells, including TGFβ, E-cadherin, and micro-RNAs, have been demonstrated to upregulate the epithelial-to-mesenchymal (EMT) transition, resulting in a more invasive phenotype [43,44].

Primary bone cancers are not the only cancers that thrive in the bone microenvironment. Many cancers metastasize to the bone because of its rich tumor-promoting environment, including breast cancer, prostate cancer, and other carcinomas [45]. Though osteosarcoma is a bone-producing tumor, various bone-metastatic breast cancers have been identified as contributing to osteolysis, or the destruction of bone tissue [46]. Enhanced production of the amino acid serine by breast cancer has been attributed to osteoclastogenesis and increased osteolysis due to bone metastases [47].

### 3.4. Osteosarcoma Predisposition

There are several genetic syndromes that predispose patients to developing osteosarcoma (Figure 2). Li-Fraumeni syndrome is caused by mutations to *TP53*, thus making it a predisposition syndrome to a number of cancers, including osteosarcoma [48]. Similarly, retinoblastoma is characterized by mutations to RB1, the retinoblastoma tumor suppressor gene, which has been identified as a driver in a subset of osteosarcoma [49]. In older adults, osteosarcoma is associated with Paget’s disease, a disease of abnormal bone recycling that results in misshapen and tumorous bones [50]. Osteosarcoma arising in Paget’s disease patients have been found to have a higher incidence of p53 mutation, as well as mutations to other tumor suppressor genes, suggesting that a second “hit” is required for osteosarcomagenesis in patients with Paget’s disease [51]. Other diseases, including Bloom syndrome (driven by a mutation to the *BLM* gene), Werner syndrome (*WRN* gene mutation), and Rothman-Thompson syndrome (*RECQL4* gene mutation), have also been correlated with increased osteosarcoma incidence [1,6,52].

Various external factors have also been identified as risk factors for osteosarcoma. As early as two and as late as 20 years after radiation therapy exposure, radiation-induced osteosarcomas have been observed; some tumors have arisen at and around radiation sites decades after initial therapy [21]. SV40 viral DNA has also been identified in as much as 50% of osteosarcoma tumors; however, there are no data to verify whether this has any causative role in osteosarcoma development [6,53,54].

## 4. Osteosarcoma Epidemiology and Diagnosis

Osteosarcoma is the most common primary pediatric and adult bone tumor [55]. Over 1000 new cases arise each year in the United States [6,17]. Approximately 80% of osteosarcomas present with a localized, primary tumor, with the other 20% presenting initially with pulmonary metastases [56]. For patients with metastatic disease, the overall survival rate is less than 20% [56]. 

Osteosarcomas are bone-forming tumors that occur primarily at the metaphysis of the bone, in regions of rapid bone growth [57]. Approximately 80% of osteosarcomas occur in the extremities, primarily in the proximal tibia, distal femur, and proximal humerus [56,58]. Clinically, most osteosarcoma patients present with pain, usually with swelling or a palpable mass identified at the site of the pain [56]. Histologic diagnosis of osteosarcoma is based on morphology as identified by radiograph [16,59]. There are six subtypes of osteosarcoma, including low-grade central osteosarcoma, osteosarcoma not otherwise specified (NOS), parosteal, periosteal, high-grade surface, and secondary osteosarcoma [57]. Within the conventional osteosarcomas are various classes of tumor based on location and originating cell, including osteoblastic, chondroblastic, and fibroblastic osteosarcomas. Osteoblastic osteosarcoma tend to make up the majority of tumors (approximately 70%); however, most osteosarcomas are genetically and morphologically heterogenous, so tumors can contain any combination of these three classes [6,16].

Depending on tumor location and stage, neoadjuvant chemotherapy with the MAP (methotrexate, doxorubicin, and cisplatin) regimen is the initial step of osteosarcoma treatment [56]. After management with resection and adjuvant chemotherapy, the cure rate of osteosarcoma is approximately 60–70% [56,58,60]. There is an association between having greater than 90% tumor necrosis after chemotherapy and overall survival [61]. Approximately 30% of patients do relapse after surgery and chemotherapy, generally within five years, at which point lung and bone metastases are the most common sites of recurrence [6,56]. 

## 5. Treatment Strategies and Molecular Targets

### 5.1. Current Standard of Care

Current treatment strategies for osteosarcoma are neoadjuvant chemotherapy with cisplatin, doxorubicin, ifosfamide, and high-dose methotrexate with leucovorin rescue, followed by surgical resection and adjuvant chemotherapy [58]. Cisplatin is an antineoplastic alkylating agent that causes DNA damage. The platinum ion in cisplatin forms bonds with DNA bases, inhibiting DNA replication and cell division [62]. Cisplatin is known to cause nerve damage, specifically leading to toxicity, but is widely used in several cancers, including lung cancer, ovarian cancer, and breast cancer, due to its efficacy [60,62]. Doxorubicin is an anthracycline, or a minor groove DNA intercalator, which also causes DNA damage by inhibiting topoisomerase II and affecting DNA replication [63]. The doses of doxorubicin given to patients are sharply regulated due to potentials for cardiotoxicity.

In the salvage setting, ifosfamide is given with etoposide [64]. Ifosfamide is a nitrogen mustard that functions as an alkylating agent that also damages DNA, thereby stopping cells from proliferating [65]. Ifosfamide creates irreparable cross links between DNA strands, which stops DNA from replicating [64]. High doses of ifosfamide are known to damage the lining of the bladder; therefore, it is often given with etoposide or mesna and has been incorporated into multidrug chemotherapy regimens with promising results [64,66]. Etoposide is a topoisomerase inhibitor that causes double stranded breaks in DNA by complexing DNA with the topoisomerase II enzyme, causing apoptosis. The combination of ifosfamide and etoposide has limited the toxicity of ifosfamide alone [64]. Ifosfamide has been approved for use in testicular cancer, osteosarcoma, soft tissue sarcoma, bladder cancer, non-small-cell lung cancer, cervical cancer, and ovarian cancer, amongst others [64,67].

Unlike cisplatin, doxorubicin, and ifosfamide, which all function by damaging DNA and inhibiting cellular division, methotrexate targets dihydrofolate reductase (DHFR), an enzyme in the folate cycle and a key metabolic component of nucleotide biosynthesis [68]. DHFR is a cellular source of tetrahydrofolate (THF), recycling THF from dihydrofolate (DHF) [69,70]. THF is required in the biosynthesis of purines and thymidylate from serine. The structure of methotrexate is similar to DHF, allowing the drug to competitively inhibit DHFR and block recycling of THF. In osteosarcoma, methotrexate is given as high doses, defined as >1 g/m^2^ [71]. In order to facilitate relatively safe use of high-dose methotrexate (HD-MTX), leucovorin rescue is used to block import of methotrexate into healthy cells. Leucovorin supplies normal cells with an additional source of THF and can thus counter the activity of methotrexate [72,73,74]. Cancerous cells lack the leucovorin transporter and are therefore susceptible to inhibition of the folate cycle by HD-MTX [75,76]. This allows the doses of HD-MTX used in osteosarcoma patients to reach doses as high as 8–12 g/m^2^. 

Even with leucovorin rescue, HD-MTX treatments still exhibit high rates of toxicity, and can lead to renal and liver failure, particularly in adults, as well as leukoencephalopathy, or damage to the white matter of the brain [68,72,77]. Due to this extremely narrow therapeutic window, an alternative—or ideally, replacement—therapeutic for HD-MTX would be beneficial.

### 5.2. Clinical Trials: The Future of Osteosarcoma Treatment

The treatment landscape for osteosarcoma in the curative setting has not evolved since the introduction of high-dose methotrexate to the standard cytotoxic chemotherapies. The palliative setting is clinical trial-rich, with over 500 clinical trials targeting novel pathways of interest in osteosarcoma. Select small-molecule inhibitor and clinically relevant immunotherapy-based clinical trials in osteosarcoma are highlighted in Table 1. Interestingly, most of the trials currently active and/or recruiting focus on combination therapies in osteosarcoma, highlighting the need for treatment strategies that exploit cellular pathways that drive specific prognostic factors in osteosarcoma, rather than neutral combinations of DNA damage agents that have activity-and side effects—across a number of cancers.

### 5.3. Targeting p53 and RB

Genetic abnormalities in *RB1* have been found in up to 70% of osteosarcoma tumors, and *TP53* mutations have been associated with approximately 90% of osteosarcoma tumors [6,22]. P53 is a tumor suppressor protein that can regulate metabolic reprogramming by acting as a sequence-specific transcription factor and altering transcription of various metabolic enzymes [78]. Mutations to *TP53* can cause structural and functional changes to the resultant mutant p53 protein, resulting in either a tumorigenic gain of function of the mutant protein, or a tumorigenic phenotype due to lack of a tumor suppressor [78]. The wild-type p53 protein has been identified as a negative regulator of osteoblasts, with p53 mutations or knockouts resulting in an increase in osteoblast function [79]. This highlights the mechanism by which osteosarcomas with mutant p53 are able to form bone. 

In some tumors that retain wild-type p53, the gene *MDM2* has been found to suppress p53 activity [80]. *MDM2* has been shown to be preferentially amplified in sarcomas, and specifically in osteosarcoma [81]. *MDM2* codes for the protein Mdm2, which is a p53 binding protein that functionally inactivates p53 transcription factor activity by binding to the N-terminal end of p53 [80,82,83]. To disrupt this binding, Nutlin-3a, an Mdm2 antagonist, is utilized to interrupt the binding of Mdm2 to p53, freeing p53 to localize to the nucleus and carry out its transcriptional function [84].

Importantly, while *TP53* and *RB* are the most common alterations in osteosarcoma, these mutations are not directly targetable in the clinic due to the complex nature of their incorporation into normal and cancerous cell biology. Mutant p53-reactivating compounds are being explored as a possible mechanism by which to selectively target mutations to *TP53*, which is encouraging for the field [85]. However, identifying and targeting the pathways driven by altered *TP53* or *RB* would allow for more directed approaches.

### 5.4. Gemcitabine and Docetaxel

Gemcitabine is a fluorinated version of the nucleoside deoxycytidine that is taken up by the nuceloside transporter SLC29A1, or human equilibrative nucleoside transporter 1 (hENT1) [86,87]. Gemcitabine incorporates into DNA as a fraudulent base pair, resulting in premature DNA strand termination [86]. Docetaxel is a mitosis inhibitor that functions by stabilizing tubulin [86]. Gemcitabine and docetaxel have been combined in multiple cancers, including soft tissue sarcoma, and have demonstrated synergy [86,88]. The efficacy of combining gemcitabine and docetaxel with other cell growth inhibitors, such as hydroxychloroquine and ADI-PEG20, in order to establish disease control is being explored in current clinical trials (NCT identifier: NCT03598595 and NCT03449901, respectively).

### 5.5. Targeting ABCB1

The p53 protein has a binding site on the promoter of ATP Binding Cassette Subfamily B Member 1 (ABCB1), a protein in a family of multidrug transporters [89,90]. ABCB1 is a drug efflux pump, making it a critical component in multidrug resistance (MDR) [91]. Mutations in these transporters have been implicated in cancer progression and drug response in a variety of cancers, including osteosarcoma and non-small-cell lung cancer, as certain single nucleotide polymorphisms (SNPs) can alter the function of the transporter [90,92]. Recent clinical trials have explored the efficacy of various chemotherapies stratified by ABCB1 expression to characterize the role of ABCB1 expression in MDR and osteosarcoma patient outcomes (NCT identifier: NCT01459484 and NCT04383288). CRISPR/Cas9 mediated inhibition of ABCB1 may also help to combat MDR in osteosarcoma [91].

### 5.6. RANK Ligand Antibodies

RANK signaling has been demonstrated to be important in osteosarcoma development, growth, and motility, and the overexpression of RANK and RANKL has been correlated with poorer outcomes [58]. Osteoblast-secreted RANKL has therefore been explored as a possible target for antibody-based therapies, as inhibiting RANK signaling on osteoclasts could decrease osteosarcoma cell migration and invasion abilities. Denosumab, an antibody to RANKL, is an anti-resorptive agent that has been used in patients with osteoporosis and is being explored in patients with refractory or relapsed osteosarcoma [9,58,93].

### 5.7. Tyrosine Kinase Inhibitors

Tyrosine kinases are enzymes such as tyrosine-protein kinase Met (c-Met) and vascular endothelial growth factor receptor 2 (VEGFR2), that phosphorylate tyrosine residues on proteins using ATP as a phosphate donor [94]. Tyrosine kinases are activated by ligand binding and have been implicated in various roles that drive the aggressive growth, migration, and invasion of osteosarcoma. As such, tyrosine kinase inhibitors have been approved in several cancers, including renal cell carcinoma, hepatocellular carcinoma, and soft tissue sarcomas. Tyrosine kinases are possible therapeutic options, though drugs that target this class of enzymes tend to be promiscuous. 

One such tyrosine kinase inhibitor, cabozantinib, inhibits c-Met and VEGFR2, in addition to other tyrosine kinases, and has been explored as a therapeutic option in osteosarcoma in multiple ongoing trials with positive results [95]. A recently completed phase II trial in Ewing’s sarcoma and osteosarcoma patients (CABONE) found that five of 42 osteosarcoma patients (12%) demonstrated a partial response to cabozantinib treatment [96]. 

Sorafenib, another tyrosine kinase inhibitor, targets extracellular signal-related kinase (ERK), VEGFR, and platelet-derived growth factor receptor (PDGFR)-α and -β. Use of single-agent sorafenib in osteosarcoma has demonstrated anti-tumor activity, but osteosarcoma has been shown to progress through sorafenib treatment for several reasons [93,97,98]. Tyrosine kinase inhibitors such as sorafenib have therefore been explored in greater detail in combination with other therapies, such as everolimus (SERIO; NCT identifier: NCT01804374) [97,99].

Regorafenib targets VEGFR2 and PDGFR-β, and tunica interna endothelial cell kinase 2 (TIE2). Regorafenib has been demonstrated to have less severe side effects than sorafenib [100]. A phase II trial in metastatic osteosarcoma (SARC024) found that regorafenib treatment had activity in progressive metastatic osteosarcoma, where progression-free survival (PFS) was doubled from 1.7 months in the placebo group to 3.6 months in the treatment group [101]. Additional phase II trials are currently recruiting to explore the efficacy of regorafenib in patients with metastatic bone sarcomas (REGOBONE; NCT identifier: NCT02389244).

### 5.8. Immune Checkpoint Inhibitors

Immunotherapies have come to the forefront in recent years as anticancer therapies [102]. One of the most successful trials implementing innate immunity involved the use of mifamurtide, an immune-stimulating compound that activates macrophages and monocytes by secreting TNFα and IL-6 [103]. Combining mifamurtide with systemic chemotherapy was demonstrated to have a significant effect on overall survival in metastatic osteosarcoma [103]. Based on these data, a phase II trial is now underway to determine if adding mifamurtide to post-operative chemotherapy has more efficacy than chemotherapy alone in high-risk osteosarcoma (SARCOME13; NCT identifier: NCT03643133). 

Programmed cell death 1 (PD-1) is a cell surface protein that is expressed on activated immune cells, including CD8+ T lymphocytes, B cells, and natural killer cells, that can also be expressed in tumor cells [102]. The ligand of PD-1, PD-L1, has also been demonstrated to be overexpressed in cancer cells and associated with poorer prognosis [104]. Camrelizumab and pembrolizumab are humanized antibodies against PD-1 and used in several cancers as immunotherapies. In a trial of pembrolizumab in patients with advanced sarcoma (SARC028; NCT identifier: NCT02301039), one of the 22 patients in the osteosarcoma arm that were given pembrolizumab demonstrated an objective response [105]. A trial of camrelizumab in combination with apatinib in osteosarcoma also seemed to slightly prolong progression-free survival, though the response rate to these therapies is as of yet not optimized [106].

### 5.9. mTOR Inhibitors

There are many alterations in the phosphoinositide 3-kinase (PI3K)/mammalian target of rapamycin (mTOR) pathway that are present in osteosarcoma, thereby suggesting mTOR as a valid target for therapy [107]. The mTORC1 signaling pathways are also activated by several oncogenes, indicating baseline activation in osteosarcoma [108]. The small molecule drug rapamycin binds to FKBP12, a receptor for immunosuppressant molecules that was found to also be a component of the protein complex that makes up mTOR complex 1 (mTORC1) [109]. Rapamycin has been utilized in osteosarcoma, but has only shown cytostatic, rather than cytotoxic, effects [108]. This is in part because rapamycin preferentially targets mTORC1, allowing mTOR complex 2 (mTORC2) to activate protein kinase B (PKB)/AKT signaling and enhance mTOR activity [110]. Various analogs of rapamycin, including everolimus and temsirolimus, have been explored in the clinic with some anti-tumoral activity (Table 1) [111]. Importantly, the adaptability of mTOR signaling in the cell allows the cell to counteract and develop resistance to mTOR inhibitors [112]. 

### 5.10. Combination Metabolic Therapies

As is the case with many cancers, common therapeutics tend to target known oncogenes, which are upregulated in many cancers. Several of the aforementioned therapeutics, conversely, are known metabolic inhibitors. Metabolism plays a key role in cancer therapeutic development as metabolic processes drive biomass production, redox homeostasis, and energy production [13]. Elevated glycolysis is a known attribute of many tumors, including osteosarcoma, as tumors require increased glucose uptake to facilitate rapid proliferation. This attribute is exploited in a diagnostic sense using 18F-FDG PET/CT [113,114]. 

The overexpression of several additional metabolic genes has also been correlated with poor survival in osteosarcoma, including X-Box Binding Protein 1 (XBP1), monocarboxylate transporter 4 (MCT4), and 3-phosphoglycerate dehydrogenase (PHGDH) [37,115,116]. Targeting these tumor biomarkers by inhibiting the overexpressed metabolic genes typically results in decreased proliferation and cytostasis, but the innate adaptability of metabolic pathways leads to limited clinical applicability of metabolic inhibitors as single agents [117,118]. As such, metabolic inhibitors must be utilized as a combination of drugs in order to cause cytotoxicity in cancer [117,119]. 

Recently, the overexpression of PHGDH, the rate-limiting enzyme of de novo serine biosynthesis, was identified in osteosarcoma [37]. Serine synthesis is particularly important for tumorigenesis as the amino acid serine contributes to protein, fatty acid, and nucleotide synthesis, as well as redox homeostasis and methylation capacity, all of which contribute to tumor growth and rapid proliferation [120]. While inhibition of PHGDH by NCT-503, a small-molecule PHGDH inhibitor, resulted in decreased proliferation, metabolic adaptations by osteosarcoma cell lines indicated elevated mTORC1 activity as a survival response. The dual inhibition of PHGDH and non-rapalog inhibition of mTORC1 resulted in significant synergistic cell death in osteosarcoma [37]. These data suggest that the use of combination metabolic inhibitors might offer a more direct, targeted approach to osteosarcoma therapy development. 

### 5.11. HER2-Targeted Therapies

Osteosarcoma cell lines have been demonstrated to have high levels of HER2 cell surface expression, and HER2 expression has been correlated with poorer outcomes and decreased response to neoadjuvant chemotherapy in osteosarcoma patients [121,122]. A recent phase I trial directing chimeric antigen receptor T (CAR T) cells, or T cells that have been specifically engineered to target a particular protein and produce an immune response, at surface-expressing HER2 showed some efficacy in osteosarcoma. 

Cell surface HER2 can also be targeted using monoclonal antibodies. Presently, a phase II trial is exploring the effects of trastuzumab deruxtecan on the treatment of HER2-positive osteosarcoma. Trastuzumab is a monoclonal antibody for HER2 that has been previously tested in combination with chemotherapy in osteosarcoma with no significant effects [123]. Trastuzumab was therefore linked to the chemotherapy drug deruxtecan, and is formulated in such a way that the chemotherapy can be directly delivered to the HER2-positive cancer cells (NCT identifier: NCT04616560).

### 5.12. Engineered Mesenchymal Stem Cells

In addition to contributing to osteosarcomagenesis and osteosarcoma progression, mesenchymal stem cells have been identified as a possible delivery system for therapeutic agents, making them uniquely exploitable for osteosarcoma treatment. Transduction of mesenchymal stem cells to express interferons (cytokine proteins that induce anti-angiogenic and anti-tumor immune activity) and interleukins allows an immune response that can be directed to the tumor site [43,103]. 

Mesenchymal stem cells can also be engineered to overexpress ligands and antibodies against the tumor that have short half-lives in the body and cannot reach the tumor site independently. For example, mesenchymal stem cells can be engineered to express tumor necrosis factor related apoptosis-inducing ligand (TRAIL), which has limited use systemically because of its short half-life, and have more significant effects on apoptosis than administering TRAIL alone [124]. Studies have also been conducted to explore the efficacy of mesenchymal stem cell-delivered TRAIL in combination with chemotherapy to further enhance these apoptotic effects [43]. 

Finally, mesenchymal stem cells can be used as a biological method to improve bone health after surgical treatment for osteosarcoma management. As mesenchymal stem cells can differentiate into the various cell types of the bone microenvironment, application of these cells to damaged bone areas has been demonstrated in several cases to be effective in filling bone defects [43,124]. Further exploration into the genetic programs that can guide mesenchymal stem cells towards one cell type over another will allow for even more targeted defect filling as an option for post-surgical therapy in osteosarcoma.

## 6. Conclusions

Though rare, osteosarcomas are the most common primary bone tumor in children and young adults. The transformation of normal functioning bone cells into osteosarcoma has been demonstrated to occur at multiple levels in mesenchymal stem cell differentiation, whereby mesenchymal stem cells can transform directly into osteosarcoma, or can undergo various stages of differentiation into osteoblasts before becoming tumorigenic. Numerous factors that are involved in osteoblast differentiation can be overexpressed or dysregulated to drive abnormal bone production and osteosarcomagenesis.

The current treatment regimen for osteosarcoma involves the use of cisplatin, doxorubicin, ifosfamide, and high-dose methotrexate. High-dose methotrexate has demonstrated significant toxicity in the liver, kidney, and brain, and novel therapies that are more specifically targeted to osteosarcoma are needed. The heterogeneity of osteosarcoma tumors necessitates that there are other potential biomarkers that could contribute to osteosarcomagenesis and be opportune targets for the development of novel chemotherapies. A critical component of cancer development, the metabolism of the tumor, offers a wide variety of upregulated pathways and overexpressed biomarkers that could encourage the development of a class of novel therapies. Elucidating the role of tumor metabolism in the progression of osteosarcoma is therefore necessary.

## Figures and Tables

**Table 1:** Current and ongoing clinical trials in osteosarcoma using molecular targeted therapies.

Identifier	Study Title	Status
NCT00470223	Combined chemotherapy with or without zoledronic acid for patients with osteosarcoma	Active, not recruiting
NCT00788125	Dasatinib, ifosfamide, carboplatin, and etoposide in treating young patients with metastatic or recurrent malignant solid tumors	Active, not recruiting
NCT01459484	ABCB1/P-glycoprotein expression as biologic stratification factor for patients with non metastatic osteosarcoma (ISG/OS-2)	Active, not recruiting
NCT01661400	Anti-angiogenic therapy post transplant (ASCR) for pediatric solid tumors	Recruiting
NCT01669369	Clinical trial of lithium carbonate combined with neo-adjuvant chemotherapy to treat osteosarcoma (Li2CO3)	Recruiting
NCT01833520	Phase I dose escalation of monthly intravenous Ra-223 dichloride in osteosarcoma	Active, not recruiting
NCT01953900	iC9-GD2-CAR-VZV-CTLs/refractory or metastatic GD2-positive sarcoma and neuroblastoma	Active, not recruiting
NCT02013336	Phase 1 study of MM-398 plus cyclophosphamide in pediatric solid tumors	Recruiting
NCT02173093	Activated T cells armed with GD2 bispecific antibody in children and young adults with neuroblastoma and osteosarcoma	Recruiting
NCT02243605	Cabozantinib S-malate in treating patients with relapsed osteosarcoma or ewing sarcoma	Active, not recruiting
NCT02357810	Pazopanib hydrochloride and topotecan hydrochloride in treating patients with metastatic soft tissue and bone sarcomas	Active, not recruiting
NCT02389244	A Phase II study evaluating efficacy and safety of regorafenib in patients with metastatic bone sarcomas	Recruiting
NCT02406781	Combination of MK3475 and metronomic cyclophosphamide in patients with advanced sarcomas: multicentre phase II trial	Recruiting
NCT02432274	Study of lenvatinib in children and adolescents with refractory or relapsed solid malignancies and young adults with osteosarcoma	Active, not recruiting
NCT02470091	Denosumab in treating patients with recurrent or refractory osteosarcoma	Active, not recruiting
NCT02484443	Dinutuximab in combination with sargramostim in treating patients with recurrent osteosarcoma	Active, not recruiting
NCT02502786	Humanized monoclonal antibody 3F8 (Hu3F8) with granulocyte-macrophage Colony stimulating factor (GM-CSF) in the treatment of recurrent osteosarcoma	Recruiting
NCT02517918	Metronomic chemotherapy in patients with advanced solid tumor with bone metastasis and advanced pretreated osteosarcoma	Recruiting
NCT02811523	In vivo lung perfusion for pulmonary metastases of sarcoma	Recruiting
NCT02867592	Cabozantinib-S-Malate in treating younger patients with recurrent, refractory, or newly diagnosed sarcomas, wilms tumor, or other rare tumors	Active, not recruiting
NCT02945800	Nab-paclitaxel and gemcitabine for recurrent/refractory sarcoma	Recruiting
NCT03006848	A phase II trial of avelumab in patients with recurrent or progressive osteosarcoma	Active, not recruiting
NCT03063983	Clinical trial evaluating metronomic chemotherapy in patients with metastatic osteosarcoma (GLATO2017)	Recruiting
NCT03277924	Trial of sunitinib plus nivolumab after standard treatment in advanced soft tissue and bone sarcomas	Recruiting
NCT03449108	LN-145 or LN-145-S1 in treating patients with relapsed or refractory ovarian cancer, anaplastic thyroid cancer, osteosarcoma, or other bone and soft tissue sarcomas	Recruiting
NCT03449901	ADI-PEG 20 in combination with gemcitabine and docetaxel for the treatment of soft tissue sarcoma, osteosarcoma, ewing’s sarcoma, and small cell lung cancer	Recruiting
NCT03478462	Dose escalation study of CLR 131 in children and adolescents with relapsed or refractory malignant tumors including but not limited to neuroblastoma, rhabdomyosarcoma, ewings sarcoma, and osteosarcoma	Recruiting
NCT03598595	Gemcitabine, docetaxel, and hydroxychloroquine in treating participants with recurrent or refractory osteosarcoma	Recruiting
NCT03618381	EGFR806 CAR T cell immunotherapy for recurrent/refractory solid tumors in children and young adults	Recruiting
NCT03628209	Nivolumab or nivolumab and azacitidine in patients with recurrent, resectable osteosarcoma	Recruiting
NCT03643133	Mifamurtide combined with post-operative chemotherapy for newly diagnosed high risk osteosarcoma patients (SARCOME13)	Recruiting
NCT03676985	A clinical study of PD-L1 antibody ZKAB001(Drug Code) in limited stage of high-grade osteosarcoma	Recruiting
NCT03718091	M6620 (VX-970) in selected solid tumors	Recruiting
NCT03742193	Pulmonary resectable metastases of osteosarcoma with apatinib and chemotherapy	Recruiting
NCT03860207	Study of the safety and efficacy of humanized 3F8 bispecific antibody (Hu3F8-BsAb) in patients with relapsed/refractory neuroblastoma, osteosarcoma and other solid tumor cancers	Recruiting
NCT03900793	Losartan + sunitinib in treatment of osteosarcoma	Recruiting
NCT03932071	Zoledronic acid in decrease the lung metastatic rate of osteosarcoma	Recruiting
NCT03960177	Glucarpidase after high-dose methotrexate in patients with osteosarcoma	Recruiting
NCT04040205	Abemaciclib for bone and soft tissue sarcoma with cyclin-dependent kinase (CDK) pathway alteration	Recruiting
NCT04055220	Efficacy and safety of regorafenib as maintenance therapy after first-line treatment in patients with bone sarcomas	Recruiting
NCT04154189	A Study to compare the efficacy and safety of ifosfamide and etoposide with or without lenvatinib in children, adolescents and young adults with relapsed and refractory osteosarcoma	Recruiting
NCT04183062	BIO-11006 for osteosarcoma and ewing’s sarcoma lung metastases	Recruiting
NCT04294511	Study of camrelizumab in combination with neoadjuvant chemotherapy in the treatment of osteosarcoma	Recruiting
NCT04351308	Comparison of MAPI + camrelizumab versus API + apatinib versus MAPI in patients with a poor response to preoperative chemotherapy for newly diagnosed high-grade osteosarcoma	Recruiting
NCT04383288	ABCB1/P-glycoprotein expression influence on non-metastatic osteosarcoma of the extremities	Recruiting
NCT04433221	Combination immunotherapy targeting sarcomas	Recruiting
NCT04483778	B7H3 CAR T cell immunotherapy for recurrent/refractory solid tumors in children and young adults	Recruiting
NCT04595994	Selinexor plus gemcitabine in selected advanced soft-tissue sarcoma and osteosarcoma	Recruiting
